# Evaluation of dormancy breaking methods for enhanced germination in four biotypes of *Brassica tournefortii*

**DOI:** 10.1038/s41598-018-35574-2

**Published:** 2018-11-20

**Authors:** Gulshan Mahajan, Navneet Kaur Mutti, Prashant Jha, Michael Walsh, Bhagirath Singh Chauhan

**Affiliations:** 1 Queensland Alliance for Agriculture and Food Innovation (QAAFI), The University of Queensland, Gatton, Queensland 4343 Australia; 2 Southern Agricultural Research Centre, Montana State University, Bozeman, MT United States; 3 Sydney Institute of Agriculture, School of Life and Environmental Sciences, The University of Sydney, Narrabri, New South Wales 2390 Australia; 4 Punjab Agricultural University, Ludhiana, 141 004 India

## Abstract

*Brassica tournefortii* is an important broadleaf weed of the winter season in the northern grain region of Australia. Knowledge of germination ecology of this weed would help in implementing effective weed control programs. A series of experiments were conducted to study the germination and dormancy behavior of four biotypes of *B. tournefortii* seeds, biotypes A (collected from barley crop), B (barley fence lines), C (chickpea crop), and D (chickpea fence lines), collected from the St George region of Queensland. The aim of this research was to determine the effectiveness of various methods on the seed dormancy release of *B. tournefortii*. Water, potassium nitrate and a soil extract did not release dormancy in *B. tournefortii* seeds (biotype A) at 20/10 °C in the light/dark regime. Cold stratification (5 °C) also did not improve germination. However, gibberellic acid (GA_3_; 100–300 mg kg^−1^) stimulated germination (>88%). Germination also improved when seeds were immersed in sodium hypochlorite (NaOCl; 42 g L^−1^) for 10 minutes and the effect was more pronounced under the complete dark environment (89% germination at a day/night temperature of 20/10 °C). The NaOCl treatment makes seeds more porous and decreases sensitivity to light. Another experiment in light/dark conducted at 25/15 °C with two biotypes (A and D) showed that, without NaOCl treatment, biotype A was more sensitive to light (29% germination) as compared to biotype D (92% germination). Our results suggest that dormancy in B*. tournefortii* seeds can be broken by the combination of NaOCl (10 min) and a dark environment. A day/night temperature of 25/15 °C was found best for optimum germination (>87%) for all the biotypes (A-D) when incubated in dark after treating with NaOCl. This research indicated a high degree of variability in germination responses for various biotypes of *B. tournefortii* seeds to various sets of conditions, which may be due to metabolic changes in response to maternal environments or genetically controlled mechanisms. Information gained from this study will be important in developing a better understanding of the dormancy behavior of *B. tournefortii* seeds in response to tillage systems or maternal environments that could influence the weed seed bank in the soil and therefore help in designing suitable weed management programs.

## Introduction

*Brassica tournefortii* Gouan. (known as wild turnip in Australia) is an important winter season broadleaf weed in the northern grain region of Australia. In Australia, it has been reported that *B. tournefortii* could reduce canola yield^1^ and other winter cereals^2^ through competition. This weed occupies the 6^th^ position in the national ranking in terms of revenue loss (AU$ 10.6 million) due to crop yield losses in Australia^2^. Some biotypes of *B. tournefortii* have developed resistance to chlorsulfuron in Western Australia and South Australia^3^.

Weeds possess unique characteristics such as outstanding adaptability, high reproducibility, resistance to abiotic stresses and germination periodicity^4^. The timing of germination is an important phenomenon in a plant’s life cycle^5^, which depends on seed dormancy mechanisms/behavior^6^. Seed dormancy is the inability of seeds to germinate in a specified period under optimal environmental conditions that otherwise are favorable for their germination^7^. Dormancy is of two types: primary and secondary dormancy. Primary dormancy is induced during the seed development phase, resulting in dormant seeds when they disperse from the mother plant. Secondary dormancy is a result of unfavorable environmental conditions after seed dispersal^8^. The existence of varying dormancy mechanisms in weed seeds enables a large proportion of the weed seed bank to emerge over an extended period of time^9,10^. Establishment of weeds in the field is strongly related to the portion of the seed bank that has been released from dormancy^10,11^. Therefore, knowledge of the seed dormancy mechanism and germination behavior of *B. tournefortii* may lead to a better design of an efficient weed management system^12^.

Seed treatment with sodium hypochlorite (NaOCl) solutions is known for reducing losses in germination caused by fungus. It has been revealed that a seed treatment with NaOCl caused changes in seed metabolism that influenced germination^13^. However, the effects of NaOCl on germination may be positive or negative. Some authors reported that NaOCl either enhances or inhibits seed germination^14,15^. On the other hand, some authors reported that NaOCl inhibited the rate of germination but not the total germination^16,17^. These differences in results might be due to differences in the duration of treatment or concentration used^18^. Therefore, these studies suggest that some weed seeds require a specific immersion time in NaOCl for releasing dormancy.

Various studies have evaluated the role of NaOCl in breaking weed seed dormancy^19–22^. These studies indicated that germination increased with increasing lengths of exposure of weed seeds to NaOCl, but up to certain limits. The immersion of weeds seeds in NaOCl made the seeds more sensitive to the exogenous GA_3_ application, light, or both^23^. Treatment of NaOCl released seed dormancy of *Avena fatua* L. possibly by modifying the properties of the hull and seed coat membranes, or by increasing the permeability of the seed to oxygen^22^.

Seed germination is an important phenomenon because the successful establishment of weeds depends on the ability of their seeds to germinate^7^. Various environmental factors influence weed seed germination, for example, germination dependent upon temperature, as the emergence rate is closely correlated with soil temperatures^24,25^. Light is another major factor influencing seed germination. Some weeds require light for germination and others prefer darkness for germination^7^. Evidence related to the ability of weed seeds to germinate at various soil depths has also been widely reported^26,27^. The optimum environmental conditions, like temperature and light, necessary for germination vary considerably, depending on weed species^28–31^. A better understanding of the germination and dormancy of *B. tournefortii* will aid in predicting its potential behavior as it spreads into new areas of Australia. Furthermore, information on the dormancy mechanism of *B. tournefortii* seeds would be useful in developing effective control measures.

Successful interruption of some *Brassica* weed seed dormancy was recorded with various methods such as alternating day/night temperatures under light/dark and dark regimes, NaOCl, and cold stratification^23,25^. A previous study in Southern Australia revealed that seeds of *B. tournefortii* had a low dormancy immediately after harvest and seed germination was not affected by light conditions^29^. However, in our study, seeds collected from the St George region exhibited a very high level of dormancy when germination was performed immediately after harvesting. These results suggest that germination behavior of *B. tournefortii* may vary with different biotypes and maternal environments. The information on the germination ecology of *B. tournefortii* seeds is limited in the northern grain region of Australia. The knowledge of seed germination ecology of *B. tournefortii* in response to the maternal environment would help in implementing weed management strategies focusing on ecological weed control. Therefore, the aim of this research was to evaluate different methods for breaking seed dormancy of *B. tournefortii* collected from the northern grain region of Australia and their effect on seed germination.

## Results

### Effects of solution/media and cold stratification

These experiments were conducted using different solutions and media to understand the dormancy mechanism in *B. tournefortii* seeds (biotype A). *B. tournefortii* seeds were unable to germinate in water when incubated in the light/dark regime at the alternating day/night temperature of 20/10 °C. Under similar conditions, complete germination (100%) was observed when seeds were treated with GA_3_ (346 mg kg^−1^); however, very little germination (1.3%) was noticed when seeds were treated with 0.01 M KNO_3_. Planting seeds in the soil extract could not stimulate germination. Similarly, leaching of seeds in water for 5 h failed to stimulate germination. Seeds also failed to germinate in the cold stratification experiments (data not shown).

### Effect of GA3 concentration on seed germination

**Figure 1:**
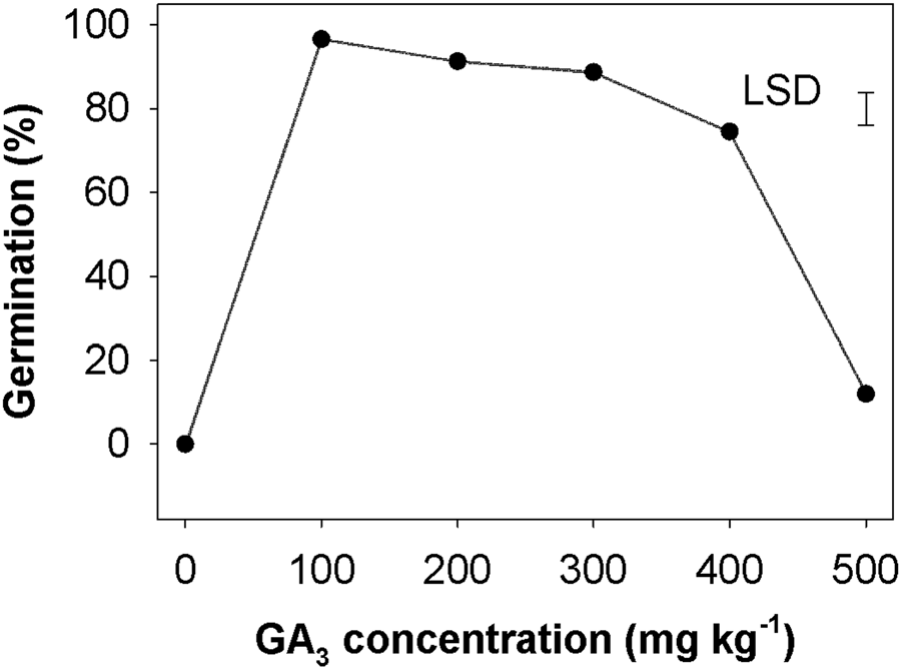
Effect of GA_3_ concentration on seed germination of *Brassica tournefortii* (biotype A incubated at 20/10 °C under light/dark).

Seeds (biotype A) were sown in GA_3_ media with concentrations ranging from 0 to 500 mg kg^−1^. No germination was found when seeds were sown in water (control) (Fig. 1). However (averaged over biotypes), seed germination was more than 88% when sown in 100 to 300 mg kg^−1^ of GA_3_. At the higher dose of GA_3_, the seed germination was reduced, and was 74.5 and 12% at 400 and 500 mg kg^−1^ of GA_3_, respectively.

### Effect of sodium hypochlorite immersion duration

**Figure 2:**
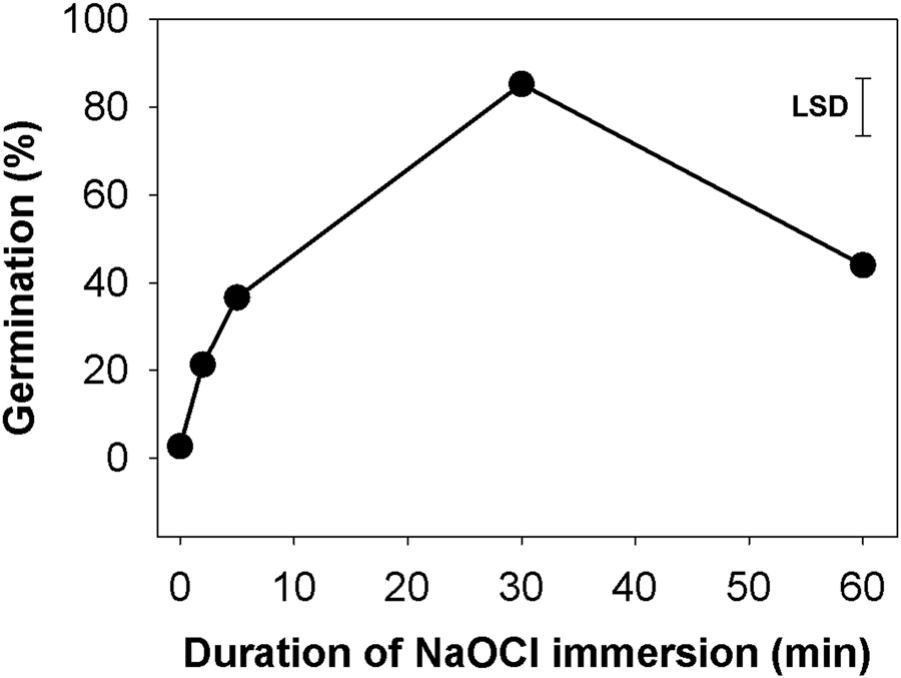
Effect of sodium hypochlorite (NaOCl) immersion duration on seed germination of *Brassica tournefortii* (biotype A) under dark at 20/10 °C.

Highest germination (85.3%) was noticed when seeds (biotype A) were immersed with NaOCl for 30 minutes (Fig. 2). Without NaOCl (only water) treatment, germination was only 2.7% and it increased slightly to 21.3 and 36.6% when seeds were immersed in NaOCl for 2 and 5 minutes, respectively. However, germination reduced with an increase in immersion duration of NaOCl from 30 to 60 minutes.

### Effect of alternating day/night temperature without NaOCl

For this experiment, two biotypes (A and D) of *B. tournefortii* were selected. In this experiment, germination (%) was influenced by the interaction of light, biotype and alternating day/night temperatures (Table 1). In the light/dark environment, germination of biotype A was not influenced by the temperature and it was highest (17%) at the alternating day/night temperature of 30/20 °C. Whereas, biotype D behaved differently under the alternating light/dark environment. In the light/dark environment, biotype D had the highest germination (88%) at the day/night temperature of 30/20 °C closely followed by 25/15 °C (71%). This biotype had 1 and 24% germination at 15/5 and 35/25 °C, respectively. In the dark environment, biotype A had higher germination (80%) at 30/20 °C compared with the other alternating temperatures. In the same light conditions, biotype D had more than 88% germination at temperatures ranging from 20/10 to 30/20 °C. Germination of this biotype was lower at the day/night temperatures of 35/25 °C (20%) and 15/5 °C (33%) compared with other temperatures (20/10, 25/15 and 30/20 °C).

**Table 1:** Effect of alternating day/night temperatures (15/5 to 35/25 °C) on the germination of biotypes A and D of *Brassica tournefortii* under light/dark (12 h photoperiod) and complete dark (24 h photoperiod).

Temperature (day/night °C)	Biotype A		Biotype D	
	Light/Dark	Dark	Light/Dark	Dark
15/5	0.0	0.0	1.3	33.3
20/10	0.0	8.0	24.0	89.3
25/15	5.3	29.3	70.7	92.0
30/20	17.3	80.0	88.0	88.0
35/25	0.0	38.7	24.0	20.0
LSD (0.05)	Temperature × Biotype × Light = 2.14			

Seeds were incubated for 14 d. Data represent the mean ± standard errors of the mean (n = 3). Seeds were not treated with NaOCl. LSD: Least significant differences.

### Effect of light, NaOCl immersion time, and GA_3_

In the light/dark regime, the highest germination was 43% when seeds (biotype A) were incubated without GA_3_ treatment after immersion with NaOCl for 30 minutes (Table 2). With GA_3_ treatment, however, the highest germination (95%) was recorded for seeds that were immersed with NaOCl for 10 min. In the dark environment, seed germination without GA_3_ treatment was highest when incubated after immersion with NaOCl for 10 min and was similar with the germination values obtained after treating with NaOCl for 20 and 30 minutes. With GA_3_ treatment, seed germination remained similar (88–97%) whether soaked (up to 30 min) or not in NaOCl solution.

**Table 2:** Interaction effects of immersion time of sodium hypochlorite (NaOCl), light and GA_3_ (100 mg kg^−1^) on seed germination of *Brassica tournefortii* (biotype A) at 20/10 °C.

Immersion time of NaOCl (min.)	Light/dark		Dark	
	Water	GA_3_	Water	GA_3_
0	0	74.0	8.0	88.8
5	0	82.0	55.3	96.7
10	3.33	94.7	89.3	94.7
20	13.3	83.3	86.0	88.7
30	43.3	52.0	83.3	88.0
60	8.0	8.0	14.7	16.0
LSD (0.05)	Light × GA_3_ × immersion time in NaOCl = 11.2			

LSD: Least significant differences.

### Effect of alternating day/night temperature and light regimes on germination

Three experiments were conducted to evaluate the interaction effects of alternating day/night temperature and light regimes on germination of four biotypes (A, B, C and D) after treating with NaOCl for 10 minutes (Tables 3 and 4). In the light/dark environment, seed germination of biotype A was highest (89%) at the day/night temperatures of 30/20 °C. However, in the dark environment, seed germination was more than 90% at the day/night temperatures of 25/15 to 35/25 °C. Biotype B also had the highest germination at the day/night temperature of 30/20 °C in the light/dark environment. In the dark regime, biotype B had >85% germination at temperatures ranging from 25/15 to 35/25 °C. In dark, biotype B had fewer germination (12%) at 15/5 °C compared with the other biotypes (81–88%). In the light/dark environment, biotype B had fewer germination (21%) at 25/15 °C than other biotypes (61–89%). In dark, at the day/night temperature of 35/25 °C, biotype C also had a fewer germination (25%) compared with other biotypes (76–94%). Overall, all tested biotypes at 25/15 °C under the dark environment had more than 85% germination.

**Table 3:** Effect of alternating day/night temperatures (15/5 to 35/25 °C) on the germination biotypes A and B of *Brassica tournefortii* weed under light/dark (12 h photoperiod) and complete dark (24 h photoperiod).

Temperature (day/night °C)	Biotype A		Biotype B	
	Light/Dark	Dark	Light/Dark	Dark
15/5	0.0	52.7	0.0	17.3
20/10	4.0	80.7	1.3	62.0
25/15	58.7	96.0	32.7	91.3
30/20	77.3	95.3	89.3	91.3
35/25	56.0	94.7	88.0	88.7
LSD (0.05)	Temperature × Biotype × Light = 12.6			

Seeds were incubated for 14 d after treating with NaOCl for 10 minutes. Data represent the mean ± standard errors of the mean (*n* = *6*). LSD: Least significant differences.

**Table 4:** Effect of alternating day/night temperatures (15/5 to 35/25 °C) on the germination of biotypes A, B, C and D of *Brassica tournefortii* weed under light/dark (12 h photoperiod) and complete dark (24 h photoperiod).

Temperature (°C)	Biotype A		Biotype B		Biotype C		Biotype D	
	L/D	D	L/D	D	L/D	D	L/D	D
15/5	0.0	81.3	0.0	12.0	22.7	88.0	4.0	88.0
20/10	0.0	97.3	0.0	72.0	52.0	90.7	6.7	94.7
25/15	61.3	98.7	21.3	86.7	89.3	89.3	72.0	100.0
30/20	90.7	98.7	81.3	98.7	82.7	72.0	84.0	98.7
35/25	45.3	94.5	53.3	93.3	50.7	25.3	77.3	76.0
LSD (0.05)	Temperature × Biotype × Light = 21.4							

Seeds were incubated for 14 d after treating with NaOCl for 10 minutes. Data represent the mean ± standard errors of the mean (*n* = *3*). L/D: Light/Dark; D: Dark.

## Discussion

Germination of *B. tournefortii* seeds was not improved in the control, soil extract and cold stratification treatments. Germination improved (>80%) when seeds were immersed in NaOCl solution for 30 minutes. Similarly, germination was stimulated with GA_3_ treatment. The application of dark treatment with or without NaOCl treatment also resulted in an increased seed germination; however, without NaOCl, the response was greater at 30/20 °C and then again reduced at 35/25 °C.

Germination was not stimulated by KNO_3_ and soil extract, indicating that soluble nutrients in the soil did not influence germination in *B. tournefortii* seeds. Contrary to this, various studies reported stimulation of seed germination in several weeds when treated with soil extracts or KNO_3_^32–34^. Similar to our results, some authors suggested that KNO_3_ had no significant effect on weed seed germination^10,35^.

We speculated that the inhibition of germination in *B. tournefortii* seeds might be attributed to germination inhibitors present in the seeds and it needs to be leached by rainfall before the seed could germinate. However, leaching of seeds with water for 5 h could not stimulate germination. This ruled out the possibility that the inhibition of germination in *B. tournefortii* was due to the presence of leachable germination inhibitors.

Dormancy in a closely related weed, *Sinapis arvensis* L., was due to the activity of growth-inhibiting substances, and as the oxygen supply reduced, the supply of these growth-inhibiting substances increased^36^. Similarly, in the present study, oxygen supply with NaOCl treatment might have increased causing a reduction in growth inhibiting substances in *B. tournefortii* seeds, which ultimately helped in releasing dormancy. These results suggest that *B. tournefortii* seeds require aeration for germination and it might be influenced by soil type, tillage operation or soil moisture in the field.

Many authors suggested that NaOCl could be used to overcome dormancy of *Sorghum halepense* (L.) Pers.^37^. The use of NaOCl increased the supply of oxygen in the seed embryo but did not remove the seed hull^22,38^. In another study, it was reported that dormancy in *Sinapis arvensis* could be overcome by acid scarification^36^. Probably, NaOCl may act and destroy the cuticle gradually and hydrolyze the walls of the epidermal cells over the entire area of the testa differently in different biotypes, thereby increasing the oxygen supply to the embryo differently for different biotypes^39,40^. NaOCl could also enhance the removal of germination inhibitors and may cause injury to the seed^20^. Various studies showed that NaOCl may induce seed germination by removing the barrier(s) to gas exchange or by oxidizing growth inhibitors in the seeds^40,41^. However, in previous studies, the interactions of NaOCl, light and temperature effects were not discussed. In our study, the failure of NaOCl alone to induce a high germination at low temperatures (15/5 and 20/10 °C) in the light/dark environment indicated that dormant seeds of *B. tournefortii* probably have two separate mechanisms for controlling dormancy. One is the germination restriction enforced by the seed coat which can be removed by NaOCl treatment. The other is the requirement for dark; germination improved in the dark when supplemented with NaOCI treatment.

In the present study, the alternating day/night temperature of 25/15 °C in the dark environment was favorable for the high germination of *B. tournefortii* seeds. In previous studies conducted in southern Australia, 20/12 °C alternating day/night temperature was found optimum for various winter weeds including Brassicaceae seeds^25,29^. However, the present study suggests that suitable temperatures for releasing dormancy in *B. tournefortii* seeds vary with the population and environmental conditions from where the seeds were collected.

Our results demonstrated that light inhibited germination of *B. tournefortii* seeds. A similar light-related response was also observed for other weeds like *Galium spurium* L.^32^. Our results suggest that *B. tournefortii* seeds may be similar to other negative photoblastic seeds in which phytochrome far-red (Pfr) remaining after seed ripening may trigger germination upon rehydration in the dark^42,43^ but again the response varied with biotypes under various alternating temperatures.

The present study revealed that darkness is the requirement for germination of *B. tournefortii* seeds; therefore, germination is expected to be low if seeds remain on the soil surface until seed death or until burial occurs through cultural operations like tillage or field preparation. Therefore, a tilled field may cause a high seedling recruitment of *B. tournefortii* seeds. These observations suggest that this weed may be a less problem in no-till farming systems, in which most seeds remain on the soil surface after crop planting.

The temperature response for germination of *B. tournefortii* seeds was different from most of the other winter weeds that usually respond at the temperature range of 20/12 °C^25,29^. Induction of dormancy by very low temperatures and high germination at 25/15 °C could make sense for this weed to have adaptation perhaps for slightly warmer conditions^44^. Therefore, it is possible that the invasion range of *B. tournefortii* may expand in high temperature zones and also outside of their main growing season. It may germinate in spring or autumn provided sufficient moisture is available. Our results suggest that the biotypes collected from northern Australia differ in their dormancy behaviour from the southern Australia biotypes^29,43^. It is quite possible that temperature or other maternal factors may have affected the dormancy mechanism in these biotypes. Therefore, it is expected that *B. tournefortii* biotypes may respond to futuristic climate change in Australia and studies on these aspects need further investigation.

Our results also demonstrated that an application of GA_3_, however, can release the dormancy of seeds in the absence of NaOCl and dark treatments. GA_3_ act as a stimulator for germination of many weeds as it enhances the mobilization of food reserves in the seeds^45^. We opined that the endogenous supply of GA_3_ as a promoter might have caused the reduction in growth inhibiting substances in *B. tournefortii* seeds and stimulated germination; however, a very high concentration of GA_3_ (500 mg kg^−1^) also inhibited the germination. GA_3_ may overcome dormancy of many seeds by changing the ratio of inhibitors and promoters within the embryo; thus, may alter the amino acid and protein syntheses in the seeds^46^. Therefore, the present results implied that GA_3_ could be used as a stimulator for germination of dormant weed seeds in the field and after germination/emergence, weed seedlings can be controlled using tillage or by suitable herbicide applications.

## Conclusions

*B. tournefortii* biotypes used in this study responded quite differently to light and/or temperature regimes tested. Dark induced more than 80% germination in NaOCl treated seeds at 25/15 and 20/10 °C. However, some variations were found with respect to biotypes, for example, greater sensitivity of biotype A to light and low temperatures (15/5 °C). GA_3_ treatment resulted in improved germination of *B. tournefortii* seeds in the light/dark environment. The NaOCI treatment made the seeds more permeable to gases and resulted in improved germination and the effect was greater in the dark environment. Without NaOCl treatment, the germination response was greater at the high day/night temperature of 30/20 °C. This study showed the importance of changes in seed coat permeability in mediating the effect of GA_3_ or light on germination and dormancy of *B. tournefortii* seeds. The results of this study also suggest that despite a high plasticity response for seed germination of *B. tournefortii* to various tested methods, the observed variations between the biotypes may ultimately be genetically controlled or due to the maternal environment from where the samples were collected. It is pertinent to mention here that the biotypes collected from northern Australia in the present study differed from the previous study conducted with southern Australia biotypes for dormancy behaviour. These observations suggest that *B. tournefortii* could be polymorphic in its response to light and temperature conditions^47^; and further studies are required to elucidate this. Differences between biotypes of *B. tournefortii* for dormancy behaviour also warrant different management practices in different regions of Australia and suggest requirements for systematic studies in this direction.

### Future thrust

The biotypes of *B. tournefortii* collected from the northern grain region of Australia behaved differently for dormancy mechanism as compared to a South Australian biotype^29^. Therefore, further studies are needed with biotypes from both regions to understand their dormancy and germination behavior. The study should also be planned to understand if the dormancy mechanism in *B. tournefortii* biotypes from different regions is due to genetic change, enzymatic change or the maternal environment.

## Materials and Methods

A series of experiments were conducted during 2017 and 2018 in the Queensland Alliance for Agriculture and Food Innovation (QAAFI) laboratory of Weed Science at the University of Queensland, Gatton Campus, Australia. For these experiments, seeds of four biotypes of *B. tournefortii* were collected from different sites situated in the Saint George region of Queensland. The coordinates for biotypes A (collected from barley crop), B (barley fence lines), C (chickpea crop) and D (chickpea fence lines) were S28^o^19.292 and E148°31.054; S27°58.549 and E149°04.572; S28°00.503 and E148°32.287; and S27°11.630, E149°03.381, respectively. Biotypes A and B were collected in October 2016 and biotypes C and D were collected in October 2017.

Seeds were collected from matured plants, threshed, and separated from the chaff. Care was taken not to damage the seed coat. The collected seed samples were dried in a properly ventilated dry place for 2–4 days to prevent microbial contamination and physiological deterioration and then stored in plastic containers in a shade house. The temperature conditions in the shade house were similar to the ambient environment but seeds were not exposed to rain. Before storing the seeds, initial germination was tested for all biotypes in the laboratory in an incubator at a fluctuating day/night temperature of 20/10 °C by following the general protocol described below. The fresh seeds of all the biotypes were found dormant (biotype A and biotype B = 0% germination; biotype C = 10% germination; biotype D = 3% germination). A series of experiments were started after 4 months of seed storage.

### Experimental setup

In each Petri dish, 25 seeds were placed on double filter papers moistened with 5 ml of different media/solution. The Petri dishes were wrapped in a polyethylene bag, sealed and then placed in an incubator at fluctuating day/night temperatures of 20/10 °C unless otherwise specified. For the dark environment, Petri dishes were wrapped in two layers of aluminum foil and then placed in the incubator. The photoperiod was set at 12 h to coincide with the high-temperature period. Fluorescent lamps were used to produce a light intensity of 115 µ mol m^−2^ s^−1^. The number of germinated seeds was counted 14 d after the start of the experiment, with the criterion for germination being a visible protrusion of the radicle.

### Effect of Germination Media and Leaching

Seed germination under different germination media was studied by incubating seeds in an incubator in the light/dark environment at 20/10 °C for 14 d. Seeds of biotype A were placed in Petri dishes using three replicates containing tap water (control), 0.01 M of KNO_3_, 346 mg kg^−1^ GA_3_, leaching in water for 5 h and 2% soil extract (10 g of soil in 500 ml water). The soil used in the experiment was a clay loam. The soil extract was used because it has been reported to stimulate germination in some weed species (e.g., *Galium spurium*)^32^.

### Effect of GA_3_

After completion of the previous experiment, another experiment was conducted using two biotypes of *B. tournefortii* (A and B) under the light/dark and dark conditions to study their germination behavior in response to various doses of GA_3_ (0, 100, 200, 300, 400 and 500 mg kg^−1^) following the general protocol.

### Effect of Cold Stratification

Seeds of biotype A were subjected to cold stratification at 5 °C for 6 wks. Seeds (*n* = 50) were placed on a double layer of Whatman No. 1 filter paper in Petri dishes and incubated in dark at 5 °C. Every week, the dishes were removed from the incubator and seeds were exhumed from the dishes. Seeds that had germinated at 5 °C were counted. The ungerminated seeds were placed on filter papers and incubated at 20/10 °C in both light/dark and dark environments. Seeds germinating at 20/10 °C were counted after 14 d of incubation and calculated as a percentage of the ungerminated seeds.

### Effect of NaOCl immersion period

The next experiment was conducted with different immersion periods of NaOCl under light/dark and complete dark conditions using the biotype A. Commercial NaOCl 42 g L^−1^ (chlorine 4% w/v, NaOH 9 g L^−1^) was used for the germination test. Seeds in required quantity were submerged in 50 ml full strength NaOCl for varying periods of 0, 5, 10, 20, 30 and 60 minutes. Seeds were washed thoroughly under tap water. They were then drained and placed in Petri dishes for germination test using the general protocol.

### Effect of NaOCl, GA_3_ and light

After the NaOCl immersion test, another germination test was performed on the biotype A to study the interaction of NaOCl immersion duration, GA_3_ (100 mg kg^−1^) and light conditions. For this, *B. tournefortii* seeds were immersed in 50 ml of NaOCl for different durations (0, 5, 10, 20, 30 and 60 minute). The treated seeds were then drained and washed with tap water. Germination test was performed using the standard protocol (with water or GA_3_) under light/dark and dark conditions.

### Effect of Temperature and Light

To determine the optimum temperature and light conditions for germination, seeds of biotypes A and B were treated with NaOCl for 10 minutes. The treated seeds were then drained, washed with tap water and 25 seeds were placed in each Petri dish that had a double layer of Whatman No. 1 filter paper moistened with 5 ml distilled water. The Petri dishes were then placed in incubators set at different alternating day/night temperatures (15/5, 20/10, 25/15, 30/20 and 35/25 °C) in light/dark and dark environments. These temperature regimes were selected to simulate temperature fluctuations during autumn and winter in the northern cropping region of Australia. This experiment was repeated with biotypes A and B and then in the third run repeated with all the four biotypes (A, B, C and D).

### Statistical Analyses

All laboratory experiments were conducted in a completely randomized design. Treatments for each experiment were replicated three times, and each experiment was repeated except the germination media, leaching and cold stratification experiments. Analysis of variance was performed on the original data obtained as a percentage germination (Elementary Designs Application, 1.0 Beta; www.agristudy.com, published by free software foundation, copyright, 2013, verified with GENSTAT 16^th^ edition; VSN International, Hemel Hempstead, UK). ANOVA’s were used to identify any significant treatment and interaction effects (P < 0.05). Where treatments were significant (P < 0.05) means were separated using Fisher’s protected LSD test at p = 0.05.
